# Health technology reassessment in the Brazilian public health system: Analysis of the current status

**DOI:** 10.1371/journal.pone.0220131

**Published:** 2019-07-29

**Authors:** Viviane Cássia Pereira, Jorge Otávio Maia Barreto, Francisco Assis da Rocha Neves

**Affiliations:** 1 Faculty of Health Sciences, University of Brasília, Brasília, Brazil; 2 Oswaldo Cruz Foundation, Brasília, Brazil; Universitat de Valencia, SPAIN

## Abstract

**Background:**

The reassessment of technologies and services offered by healthcare systems is recent initiative and still without a widely adopted and evaluated method. To a better understanding of this process in Brazil, we have described the health technology reassessment (HTR) performed by the National Committee for Health Technology Incorporation (Conitec) into Brazilian public health system (SUS).

**Methods:**

A documental, exploratory, descriptive, retrospective study with qualitative-quantitative approach regarding the HTR performed by Conitec from January 2012 to November 2017.

**Results:**

After applying the criteria of inclusion and exclusion, we selected 47 technologies for this study. The vast majority of the demands (41 demands) came from the public sector, and only six from the private sector. Most of the requests referred to the exclusion of specific indication; followed by extension of use, withdraw of the technology from SUS, maintenance, and restriction of use. The dimensions of analysis found in the recommendation reports were scientific evidence on efficacy, effectiveness and safety, disease-related issues, issues related to the use of technology, costs, and social participation. However, these dimensions were not included in all analysis, and a standardized structure of the reports has not been observed. The most relevant decision factors considered for decision-making were efficacy, safety and use of the technology.

**Conclusion:**

During a six-year period of Conitec actuation, we could find some reassessments of technologies that are available in SUS. We observed that these activities had enabled progress, however, they are still not yet structured, with gaps in the selection process, and the assessment since no methodology and criteria for proper conduct were established.

## Introduction

The process of Health Technology Assessment (HTA) considers primarily the results of efficacy studies, mostly from randomized clinical studies. However, despite being essentials, these studies often point out a high degree of uncertainty to the real benefits to the population, which, in many cases, is not proportional to the resources invested [1,2]. This phenomenon is easily observed, for example, in new technologies for oncological treatments, which costs are incredibly high and the benefits are marginal [3].

According to the Brazilian Institute of Geography and Statistics (IBGE), between 2013 and 2014, the final consumption of health goods and services rose from 8.2% to 8.7% of Gross Domestic Product (GDP). In 2015, the health expenditures were R$ 546 billion (9.1% of GDP), with 79.2% of health services and 19% of medicines. The rest referred to the consumption of other medical, ophthalmological, and dental materials. Considering these data, approximately one-fifth of health expenditures is related to the use of medicinal drugs, only surpassed by hospital cost, outpatient and preventive services and sanitary surveillance [4].

It is essential to define tools that can support decision-makers in the adoption and re-evaluation of technologies within health systems. Among many reasons for this approach, we can cite the prospects for an increase in the use of drugs and other health technologies to meet the demands of a population that are progressively expanding associated with an increase in the prevalence of chronic degenerative diseases, together with the highly complex and contextualized analysis for selection of technologies and services.

Regarding the adoption of new technologies, there has been much effort in the study of methods used by Health Technology Assessment (HTA) agencies and how to improve them, so that this process can apprehend all the relevant aspects of the intervention in a given context. Consequently, more objective and transparent methodologies and criteria that take into account the perspectives of different stakeholders have been developed and validated in several countries to optimize the allocation of resources and contribute to more consistent decisions before society [5–7].

On the other hand, studies on reassessment methodologies to monitor and evaluate the use of technologies are less frequent and have only recently become available; more precisely, since 2006 [8]. The health policy forum of the HTAi (Health Technology Assessment International) of 2012 proposed that health reassessment of technology (HTR) become an HTA standardized practice. HTR is defined as "a structured, evidence-based assessment of the clinical, social, ethical, and economic effects of a technology currently used in the healthcare system, to inform optimal use of that technology in comparison to its alternatives" [9].

Such a process is fundamental to the quality of care offered to the patient and the sustainability of the health system, as resources could be reinvested in more useful or less expensive health services. Within the technology lifecycle, this phase aims at finding technologies that present inferior results to those that subsidize their adoption or even interventions that are ineffective or harmful to the patient or the health service [10]. Hence, the HTR, recommendations such as maintenance, total disinvestment, partial disinvestment (restriction of the user group), extension of use, reduction of quantity and price can be adopted [11].

Some studies were conducted targeting low-value practices, which, according to Colla and colleagues, are "the use of care that is unlikely to benefit the patient given the cost, available alternatives, and preferences of the patient" [10]. Prasad and colleagues evaluated 363 studies on established health interventions and observed that 138 practices (38.0%) confirmed their efficacy, 79 (21.8%) presented inconclusive data and 146 (40.2%) were ineffective [11].

In another review, Niven and colleagues have reported cardiovascular diseases, arthritis, and menopause as the most common target conditions in de-adoption studies–discontinuation of current clinical practice. The most researched therapies were cyclooxygenase-2 inhibitors (COX-2) and other non-steroidal anti-inflammatory drugs (NSAIDs), hormone replacement therapy and percutaneous coronary intervention [12].

Considering that the low-value practices are a potential waste and can be harmful to the patient’s health, some countries (e.g.: France, England, Australia, Spain, Canada, and the United Kingdom) have already begun monitoring the technology after a certain period past its implementation in the health care system [13–16].

During the French health system reform, there was a review of the complete list of reimbursed drugs and those considered ineffective were withdrawn from the health system. In addition to this strategy, there were also others like greater access to average European prices for innovative drugs, stimulation of discounts for over-prescription drugs and programs to increase the prescription of generic medicines. The result of HTR and the further decision not to reimburse the ineffective medications represented an estimated savings of 450 million euros/year, in addition to the savings generated by the implementation of the other reform initiatives (1.7 billion euros / year—period from 2003 to 2007) [13].

These data about the French health system emphasize that combining several strategies to improve technologies management, results in a significant impact on economic resources. However, the sustainability of health systems involves other aspects beyond the financials ones (structural analysis); such as the continuity of the provided services (organizational analysis) and the obtained benefits (individual analysis) [17]. These aspects must be audited and assessed continuously to ensure waste reduction, without compromising the health benefits already gained, plus helping to foster improvements.

In Brazil, the National Committee for Health Technology Incorporation (Conitec) was established in 2011 to improve the decision-making process in HTA. The primary purpose of this committee is advising the Ministry of Health on decisions related to the adoption, disinvestment or changes in the use of health technologies in SUS, as well as the development or update of clinical protocols or therapeutic guidelines [18]. Conitec chief responsibility is to issue a recommendation based on efficacy, accuracy, effectiveness, and safety of the technology, comparative economic evaluation of the benefits and costs concerning the technologies already available in SUS and budget impact. However, to issue the final decision is the duty of the Secretary of Science, Technology and Strategic Inputs of the Ministry of Health [18,19].

Conitec creation has promoted advances in the institutionalization of HTA in the Brazilian health system, due to the rationality and use of clinical and economic evidence in decisions about the inclusion of new technologies in SUS. Some studies on the work of the Committee have been published with the purpose of outlining the evaluation process, the demands, and the recommendations about the adoption of technologies [10–12]. However, such studies analyzed the set of evaluations and recommendations, without highlighting the specific characteristics of the HTR process used by Conitec.

Therefore, the present study aimed to analyze and characterize the process of HTR carried out by Conitec, focusing on the dimensions of analysis, criteria and decision factors that were relevant for the Committee recommendations.

This study is the first step of our project. The full project consists of 5 steps: 1- Review of the current state of HTR carried out by Conitec; 2- Systematic review of the literature; 3- Development of a RTS framework; 4- Pilot study using the RTS framework; 5- Evaluation of the results, adjustments and validation of the RTS framework.

## Materials and methods

We carried out a documental, exploratory, descriptive, retrospective study with qualitative-quantitative approach regarding the HTR performed by Conitec from January 2012 to November 2017.

The study comprised the following steps:

- Selection and classification of technologies;
- Data extraction;
- Data analysis.

### Primary outcomes

Description of the HTR process and Conitec recommendations from the analysis of (i) consistency in the report on presenting analysis of dimension and criteria as evidence, issues related to the context of disease and technology, costs, public consultation, and others–and (ii) the decision factors for Conitec recommendations.

### Classification and selection of technologies

Most of the final decisions were issued as “adoption” or “no adoption” of technology, regardless of the technology is already available in n SUS. Thus, for selecting the reassessed technologies, the types of evaluation requests, and so the final decisions, were reclassified based on the availability of the technology in SUS for the assessed indications, as shown in the Table 1.

**Table 1:** Definition of the types of request/decision to classify the technologies evaluated by Conitec.

Types of request	Definition
a) Adoption of new health technologies	a.1) Technology is not available in SUS during the assessment period. a.2) New concentration, new pharmaceutical form and new route of administration: presentation not available in SUS during the assessment period.
b) Extension of use	b.1) New indication: extension of use for an indication different from those already approved for use within the SUS. b.2) Change in treatment line (e.g., request to change the drug indication from 2nd line to 1st line); b.3) Widening in the age range; b.4) Increase in the period of treatment: increase in the use of technology for the same indication for which it was already available in the SUS.
c) Maintenance	Maintenance of the technology for the same indication for which it was already available in SUS.
d) Restriction of use	Restriction of the group of patients using the technology.
e) Exclusion of indication	Exclusion of specific indication for the use of technology but maintaining its use for the other indications approved within the SUS.
f) Exclusion from SUS (Delisting)	Exclusion of technology from SUS.
g) Exclusion of the International Classification of Diseases (ICD) from the protocol	Exclusion of specific indication from the therapeutic protocol.
h) Protocols/Guidelines approval	Approval of update or new Clinical Protocol and Therapeutic Guideline.

#### Inclusion criteria

All technologies available in SUS, and that were reassessed for an indication already approved within the health system.

- Health technologies: drugs, vaccines, health devices, and procedures.
- Reassessed technologies: health technologies (already) available in SUS, assessed in the same presentation and for the same indication.

The reassessment request/decision was an extension of use/not extension of use (items b.2, b.3, b.4); maintenance/delisting from SUS (items c and f), restriction of use (item d); maintenance/exclusion of indication (item e).

#### Exclusion criteria

The exclusion criteria were as follows:

- Clinical protocols and therapeutic guidelines were not included. Each inclusion, exclusion, and change of use of technologies when drawing up or revising the protocols go through the same process of evaluation of other technologies and are contained in the set of technologies evaluated [20].
- New health technologies (in SUS context): technologies that were not available in SUS during the assessment period.
- Drugs with new concentration, new pharmaceutical form and new route of administration were also considered as new technologies for the health system, and, therefore, excluded from the present study.
- Technologies with a new indication, although already available in the health system for other clinical situations.

### Search and data extraction methods

The public data were collected directly from the recommendation reports available on the Committee website (January 2012 until November 2017) [21], namely:

- Technology name;
- Amount of technologies by decision: the analysis unit was defined as being the reassessed technology, even in cases where the demand or decision was related to more than one technology;
- Type of technology: drug, vaccine, device, procedure;
- Type of applicant: public sector (Ministry of Health and related institutions, State and Municipal Health Secretariats, Judicial Power) and private sector (pharmaceutical laboratories, nonprofit organizations as patient associations, medical organizations/associations);
- Indication;
- Type of assessment (reclassification as described in the method): new technology, new presentation, extension of use (new indication), extension of use (a change in the therapeutic line, wide age range, increase in the treatment period), maintenance, restriction of use, exclusion of indication, delisting, exclusion of the International Classification of Diseases (ICD) from the protocol, protocol/guideline approval;
- Dimensions of analysis and criteria in the recommendation report;
- Decision factors for the recommendation; and
- Final decision.

The data related to the dimensions of analysis and criteria were extracted considering the main items in recommendation reports. The data related to the decision factors were collected from the items “final considerations” and “final recommendation” when the reports presented them. For reports that did not have a standardized structure, the above information was extracted from the full reading of the reports.

### Data analysis

Statistical analysis was performed using the frequency of the dimensions of analysis, criteria and decision factors presented in the recommendation reports. Then the findings were organized in tables with narrative description and discussion.

## Results

### Description of HTA requested to Conitec

**Fig 1:**
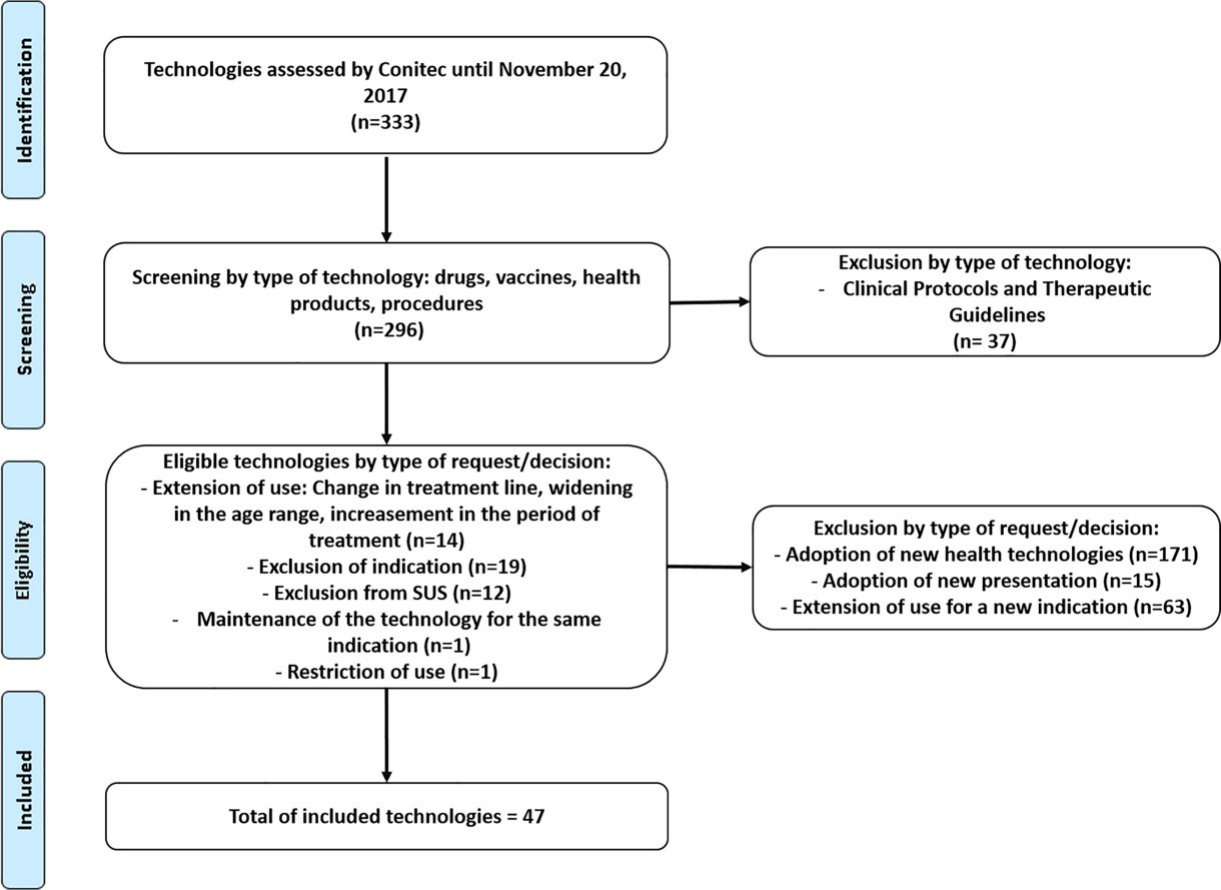
Flow diagram through the phases of selection of the technologies reassessed by Conitec.

Conitec had assessed 333 technologies, which includes drugs, vaccines, devices, procedures, and clinical protocols from January 2012 until November 2017 (S1 Table). Among of them, the vast majority was related to the adoption of new technologies (55.8%), followed by analysis of new indications for technologies already offered by the health system (19%) and approval of clinical protocols (11%). Other requests accounted for 14.2% (Fig 1) of the demands.

After applying the inclusion and disinvestment criteria described in the method, 47 health technologies were selected (14.1%), whose requests/decisions referred to a reassessment (Fig 1).

Among the 47 technologies included in the study, 44 (93.6%) were drugs, two (4.2%) procedures and one vaccine (2.1%). Regarding the type of claimant, 41 (87%) demands originated from the public sector and only six (12.8%) from the private sector. The public demands–from the Ministry of Health and affiliated institutions–related to the extension, restriction, maintenance of use, exclusion of indication or complete delisting from SUS. All these public requests had a favorable recommendation, except the demand for widening the age range for mammography as a breast cancer screening method, which the judgment was unfavorable. All the private demands from pharmaceutical laboratories were to increase the use of the medication; three of them had received a favorable decision and three, unfavorable.

Regarding the reason of the requests, the exclusion of some specific indication (n = 19; 40.4%) was the most mentioned reason. In such cases, the technologies remained in the health system for the previously approved use. Fourteen requirements (29.8%) were made to the extension of the use of a given technology, twelve (25.5%) to delisting from SUS and maintenance and restriction, a request each (2.1%).

Rheumatoid arthritis, HIV/AIDS, and hepatitis C were the indications for which most of the technologies were reassessed with seventeen (36.2%), ten (21.2%) and six (12.8%) demand, respectively. The other indications were Crohn's disease (two), multiple sclerosis (two), hepatitis B (two), breast cancer (one) epidermoid carcinoma (one), cystic fibrosis and exocrine pancreatic disease (one), Gaucher disease (one), hepatitis A (one), malaria (one) and congenital syphilis (one).

### Dimensions of analysis in Conitec recommendation reports

The dimensions of analysis and the criteria for organizing the results were defined based on the information collected. Then, the data were structured according to the dimensions presented in the reports: context regarding the disease and technology, scientific evidence, costs, and social participation (Tables 2 and 3).

**Table 2:** Dimensions of analysis, criteria and decision factors according to Conitec recommendation reports.

Dimensions							Context					Scientific evidence	Costs		Social participation
					Criteria		Context of disease	Technology implementation	Use of technology	NG	ID	EFC, EFT and/or SAF	EA	BI	Public consultation
**Technology**	**Amount of technologies**	**Type of technology**	**Applicant**	**Indication**	**Reason for the request**	**Decision**	** **	** **	** **	** **	** **	** **	** **	** **	** **
Abatacept	1	Drug	Private	Rheumatoid arthritis	Extension of use^a^	Unfavorable	**√**	** **	** **	** **	**√**	**√** **DF: EFC, SAF**	**√**	**√**	**√**
Adalimumab, certolizumab, etanercept, infliximab, golimumab, rituximab, abatacept and tocilizumab	8	Drug	Public	Rheumatoid arthritis	Exclusion of indication	Favorable	** **	** **	** **	**√**	** **	**DF: EFC**	** **	** **	** **
Adefovir	1	Drug	Public	Hepatitis B	Exclusion from SUS	Favorable	**√**	** **	** **	** **	** **	**√** **DF: EFC, SAF**	** **	** **	** **
Interferon alfa-2b	1	Drug	Public	Hepatitis B	Exclusion of indication	Favorable	**√**	** **	**DF: adherence**	** **	** **	**√** **DF: EFC, EFT, SAF**	** **	** **	** **
Taliglucerase alfa	1	Drug	Public	Gaucher disease	Extension of use^b^	Favorable	**√**	**√** **DF: extension of registration**	** **	** **	** **	**√** **DF: EFC**	** **	** **	** **
Artemether	1	Drug	Public	Malaria	Exclusion from SUS	Favorable	**√**	** **	** **	** **	** **	**√** **DF: EFC, SAF**	** **	**√**	**√**
Interferon beta—intramuscular	1	Drug	Public	Multiple Sclerosis	Restriction of use	Favorable	**√**	** **	**√** **DF: adherence**	** **	** **	**√** **DF: EFC, EFT**	** **	** **	**√**
Ciclosporin	1	Drug	Public	Crohn Disease	Exclusion of indication	Favorable	**√**	** **	** **	** **	** **	**√** **DF: EFC**	** **	** **	** **
Ciclosporin	1	Drug	Public	Rheumatoid arthritis—specific cases	Exclusion of indication	Favorable	** **	** **	** **	**√**	** **	**DF: EFC**	** **	** **	** **
Dolutegravir	1	Drug	Private	HIV/AIDS	Extension of use^a^	Favorable	**√**	** **	** **	** **	**√**	**√** **DF: EFC, SAF**	**√**	**√**	**√**
Dolutegravir and darunavir	2	Drug	Public	HIV/AIDS	Extension of use^a^	Favorable	**√**	** **	**DF: adherence**	** **	** **	**√** **DF: EFC**	** **	**√**	** **
Stavudine and indinavir	2	Drug	Public	HIV/AIDS	Exclusion from SUS	Favorable	** **	** **	**DF: obsolescence**	**√**	** **	**DF: EFC, SAF**	** **	** **	** **
Filgrastim and epoetin alfa	2	Drug	Public	Hepatitis C	Exclusion of indication	Favorable	**√**	** **	**DF: obsolescence**	**√**	** **	**√**	** **	** **	** **
Fingolimod	1	Drug	Private	Multiple Sclerosis	Extension of use^a^	Favorable	**√** **DF: UMN**	** **	** **	** **	**√**	**√** **DF: EFC**	**√**	**√**	**√**
Fosamprenavir and didanosine	2	Drug	Public	HIV/AIDS	Exclusion from SUS	Favorable	** **	** **	**DF: adherence; obsolescence**	**√**	** **	**DF: EFC, SAF**	** **	** **	** **
Leflunomide, chloroquine, hydroxychloroquine, methotrexate and sulfasalazine	5	Drug	Public	Rheumatoid arthritis—specific cases	Exclusion of indication	Favorable	** **	** **	** **	**√**	** **	**DF: EFC**	** **	** **	** **
Mammography	1	Procedure	Public	Breast Neoplasms	Extension of use^b^	Unfavorable	**√**	** **	** **	** **	** **	**√** **DF: EFC**	** **	** **	**√**
Mesalazine	1	Drug	Public	Crohn Disease	Exclusion of indication	Favorable	**√**	** **	** **	** **	** **	**√** **DF: EFC**	** **	** **	** **
Molgramostim	1	Drug	Public	Various	Exclusion from SUS	Favorable	** **	**√** **DF: lack of registration**	**√** **DF: obsolescence**	** **	** **	**DF: SAF**	** **	** **	** **
Pancrelipase	1	Drug	Public	Cystic fibrosis and exocrine pancreatic Insufficiency	Exclusion from SUS	Favorable	** **	**√** **DF: lack of registration**	**√**	** **	** **	** **	** **	** **	** **
Benzathine penicillin	1	Drug	Public	Congenital Syphilis	Maintenance	Favorable	**√**	** **	** **	** **	** **	**√** **DF: EFC**	** **	** **	**√**
Adjuvant chemotherapy	1	Procedure	Public	Squamous cell carcinoma	Exclusion from SUS	Favorable	**√**	** **	** **	** **	** **	**√** **DF: EFC**	** **	** **	** **
Raltegravir	1	Drug	Public	HIV/AIDS	Extension of use^b^	Favorable	**√**	**√** **DF: extension of registration**	**DF: adherence**	** **	**√**	**√** **DF: EFC, SAF**	** **	**√** **FD**	** **
Raltegravir	1	Drug	Private	HIV/AIDS	Extension of use^a^	Favorable	**√**	** **	** **	** **	**√**	**√** **DF: EFC, SAF**	**√**	**√** **FD**	**√**
Ritonavir	1	Drug	Public	HIV/AIDS	Exclusion from SUS	Favorable	** **	**√** **DF: thermolability**	** **	** **	** **	** **	** **	**√**	** **
Sofosbuvir and daclatasvir	2	Drug	Public	Hepatitis C	Extension of use^c^	Favorable	**√**	** **	**√** **DF: adherence**	** **	** **	**√** **DF: EFC, EFT, SAF**	** **	**√** **FD**	**√**
Telaprevir and boceprevir	2	Drug	Public	Hepatitis C	Exclusion from SUS	Favorable	**√**	** **	**√** **DF: adherence; obsolescence**	**√**	** **	**√** **DF: EFC, EFT, SAF**	** **	**√** **FD**	** **
Tocilizumab	1	Drug	Private	Rheumatoid arthritis	Extension of use^a^	Unfavorable	**√**	** **	** **	** **	** **	**√** **DF: EFC**	**√**	**√** **FD**	**√**
Tocilizumab	1	Drug	Private	Rheumatoid arthritis	Extension of use^a^	Unfavorable	**√**	** **	** **	** **	** **	**√** **DF: EFC, SAF**	**√**	**√** **FD**	**√**
Hepatitis A vaccine	1	Vaccine	Public	Hepatitis A	Extension of use^b^	Favorable	**√** **DF: UMN**	**√** **DF: logistics of the program**	**√** **DF: vaccine utilization**	** **	**√**	**√** **DF: EFC, EFT**	**√** **DF: CE**	**√** **FD**	**√**

Check mark (“✓”) indicates the presence of the information and FD (factor decision) indicates the determinant criteria for the decision-making. BI: Budget impact; DF: Decision factors; EA: Economic evaluation; EFC: Efficacy; EFT: Effectiveness; ID: International documents; NG: National guideline; SAF: Safety; UNM: Unmeet need.

^a^Extension of use—change in the therapeutic line

^b^Extension of use—wider age range

^c^Extension of use—increase in the treatment period.

**Table 3:** Synthesis of the dimensions of analysis, criteria and decision factors according to Conitec recommendation reports.

Criteria	Number of technologies that presented information concerning the criteria	Number of technologies for which the criterion was considered a decision factor
**DIMENSION: CONTEXT**		
**Disease**	26	2
**Recommendations/national guidelines**	22	0
**Use of technology**	8	15
**Technology implementation**	6	6
**International documents**	6	0
**DIMENSION: SCIENTIFIC EVIDENCE**		
**Efficacy**	26 efficacy, safety or effectiveness	42
**Safety**		17
**Effectiveness**		7
**DIMENSION: COSTS**		
**Budget impact**	16	9
**Economic evaluation**	7	1
**DIMENSION: SOCIAL PARTICIPATION**		
**Public consultation**	13	0

A little more than half of the evaluations (n = 26; 55.3%) addressed aspects related to the disease, like its evolution, epidemiological data, diagnosis and treatment offered by the health system. The amount of technology used within the public system was taken into consideration in eight cases (17%). Issues concerning the implementation of technology as the availability of the technology in the Brazilian market, drug thermolability, storage and logistics for distribution and changes on the indications approved by the National Agency of Sanitary Surveillance–ANVISA were considered only in a few analyses (n = 6; 12.7%).

Clinical protocol development or updating had subsidized decisions on 22 technologies (46.8%), all relative to the exclusion of specific technology indication or delisting from SUS. International documents as reports of HTA and clinical protocols had based decisions on an extension of use (change in the treatment line or wide age range) of six technologies (12.8%).

Scientific evidence about efficacy, effectiveness or safety were presented in 26 technologies assessment (55.3%). All the reports without scientific evidence were related to decisions of exclusion of the technology from SUS or exclusion of specific indication for a technology. The justifications for these cases were as follows: adjustments in the clinical protocol (14 technologies) with wrong recommendations (drugs recommended to treat conditions without indication in the package insert or without data from scientific literature); replacement of obsolete drugs (seven technologies), unavailability in the market (two technologies), stability/storage (one technology). In nineteen cases that the scientific evidence was lacking, the arguments supporting the decision were related to less efficacy or safety, along with others factors, however, no scientific data were presented in the reports.

Of all assessments, clinical results extracted from the SUS database were used only in comparing the performance of interferon beta in the treatment of multiple sclerosis. Follow-up data of around ten years of patients using interferon beta for the treatment of multiple sclerosis in SUS demonstrated a statistically lower performance for intramuscular interferon beta as compared to another interferon beta regarding effectiveness (relapses and death outcomes) and treatment adherence.

The budget impact estimates supported 16 decisions (34%)—twelve for extended use (increasing treatment period, changing lines or age range) of HIV/AIDS drugs, rheumatoid arthritis, multiple sclerosis, hepatitis A and hepatitis C and another four to an exclusion from the SUS of technologies for HIV/AIDS, hepatitis C and malaria. Seven of these assessments (15%) also used economic studies; being three cost-minimization studies, three cost-effectiveness study, and one a cost-efficacy comparison. All these seven evaluations were related to the requests for extension of use–six cases were for a change treatment line of drugs for rheumatoid arthritis, multiple sclerosis and AIDS, and one for the widening the age range for hepatitis A vaccine.

In what concerns the public consultation, thirteen (27.6%) of the assessed technologies had undergone this phase.

### Decision factors for Conitec recommendations

Although various information was taken into account for Conitec deliberation, some criteria were regarded as crucial for the decision-making (Tables 2 and 3). Concerning the disease, the justification for unmet need (lack of an option in SUS) was fundamental for the use of the drug fingolimod for multiple sclerosis (option for a failure of first and second line drugs) and the hepatitis A vaccine (child population not covered by the vaccine).

Questions of adhesion, including dosage, route of administration and tolerability, impacted in the evaluations for restriction of use of intramuscular interferon beta, expansion of use of dolutegravir, darunavir and raltegravir, and exclusion of the medicaments fosamprenavir, didanosine, telaprevir and boceprevir from SUS list and exclusion of indications for the use of injectable interferon alfa-2b in the treatment of hepatitis B.

In some delisted drugs, like telaprevir, boceprevir, stavudine, indinavir, and didanosine obsolescence was perceived, and more efficient and safer drugs substituted them. The medicament molgramostim, that was not marketed in Brazil due to its adverse events, was also delisted. The obsolescence was also verified in the evaluation of some specific indications of filgrastim and epoetin alfa–situations of complications with the use of telaprevir and boceprevir. With the exclusion of telaprevir and boceprevir, filgrastim and epoetin alfa lost their utility for this indication, although they remained in SUS for other conditions.

For most technologies (42, 89%), the criterion of efficacy was essential for the Committee to decide on extension of use (fourteen technologies), exclusion of specific indication for the use of a technology (seventeen technologies), delisting from SUS (nine technologies), maintenance in the health system and restriction of use (one technology each).

The fourteen drugs for which the efficacy was essential for decision of extension of use were, related mainly to HIV/AIDS, rheumatoid arthritis and hepatitis C (5, 3 and 2 technologies, respectively). The other indications were: Gaucher disease, multiple sclerosis, breast neoplasm, and hepatitis A. In the cases of exclusion of indication (17 drugs), this criterion was used majority for drugs for rheumatoid arthritis treatment (14 drugs). At last, the efficacy of drugs for HIV/AIDS (5 drugs) and hepatitis C (2 drugs) was relevant for the de-listing decision-making. So, the relevance of this criterion was observed mainly for drugs related to HIV/AIDS, rheumatoid arthritis and hepatitis, independent of the kind of recommendation.

Five technologies did not consider efficacy as a deciding factor for the recommendation. These drugs were related to exclusion from the SUS (Three drugs for HIV/AIDS, cystic fibrosis and various indications) and exclusion of indication (Two drugs for hepatitis C).

Data effectiveness from the scientific literature and the SUS data were relevant in seven assessment for extension, exclusion, and restriction of use. To evaluate the comparison of the efficacy of interferon beta, we used data from SUS that resulted in the restriction of intramuscular beta interferon use. For the extension of the treatment period with sofosbuvir and daclatasvir, real-world evidence obtained from European patient cirrhotic genotype 3 who received treatment for hepatitis C during 24 weeks showing higher therapeutic successful results rates were essential [22,23]. Due to this evidence, the most important therapeutic guides in the world had started to recommend the extension of 24 weeks for patients’ cirrhotic genotype 3 treated with sofosbuvir in association or not to the ribavirin. In the case of hepatitis C, telaprevir and boceprevir were withdraw from SUS due to the adoption of sofosbuvir, daclatasvir and simeprevir for the treatment of chronic C viral hepatitis, which was considered more effective and safer according to the scientific literature. In the hepatitis vaccine assessment, the effectiveness data in some countries had also been relevant for widening the age range. Exclusion of interferon alfa-2b from the list of technologies for hepatitis B also considered effectiveness data to be replaced by peginterferon alfa.

Information about safety supported the extension of the use of seven technologies, decisions about the exclusion of nine technologies from SUS and exclusion of one specific indication. The extensions of use referred to the change of line (raltegravir, abatacept, tocilizumab and to dolutegravir), increase in the treatment period (sofosbuvir and daclatasvir) and the increase of age group (raltegravir). In general, the delisted technologies were replaced by others, to maintain options in SUS, so, patients were not uncovered (artemether, fosamprenavir, didanosine, adefovir, telaprevir, boceprevir, molgramostim, stavudine, and indinavir).

The costs were relevant for nine recommendations. The budget impact was considered a decision factor in seven decisions about the expansion of use–raltegravir (two decisions), tocilizumab (two decisions), sofosbuvir, daclatasvir and hepatitis A vaccine–and in the exclusions of telaprevir and boceprevir from SUS. The economic evaluation was taken into account to support the extension of use of hepatitis A vacccine.

Other information were used to ratify the Committee's recommendations, such as the expansion of indications for registration with the ANVISA (Taliglucerase alfa and raltegravir), lack of registration in Brazil (Pancrelipase and molgramostim), logistics of the program (Hepatitis A vaccine) and thermolability (Ritonavir).

## Discussion

In order to analyze and describe the process of HTR performed by Conitec, 47 technologies were selected. Several requests for reassessment originated in the Ministry of Health, Health Departments and other institutions that comprise SUS, aimed at adapting the technologies within the health system. There were a few private requests—a sixth as compared to the public, and all were to change the treatment line for which the technology was already being offered by the SUS. Therefore, the private requirements did not aim to fully analyze the interventions within the system, but extend it use to a higher number of people.

There was only one decision, to the public demands, contrary to the request for widening the age range for mammography as a method of breast cancer prevention because of the uncertainty benefits to the population. For a private application, half of the deliberations was favorable and half-unfavorable to the extension of use.

Yuba et al. study evaluated the recommendations of all Conitec evaluations from 2012 to 2016 [24] and also noted that, proportionally, more assessments were issued as favorable when the applicant was the public sector in detriment of the private sector. Thus, there is a tendency that public requests obtain favorable recommendations. This phenomenon deserves thorough scrutiny about its causes, which could be linked, among other hypotheses, to the source of demand once the public claimants would be more aligned to the real needs of the health system or because it could occur a biased assessment to such requirements based on the relationship between plaintiff and appraiser.

Regarding the reason of the requests, the exclusion of some specific indication was the most mentioned reason, followed by extension of the use of a technology and delisting from SUS. These results reflect a worldwide trend in using HTR, which is not focusing on the total exclusion of technology, but rather on refining clinical indications to optimize technology usage and the resources allocation within a healthcare system. In the UK, The National Institute for Health and Clinical Excellence is going towards this direction, having concluded that in practice, few technologies are prone to total disinvestment [14]. In New Zealand, due to the excellent potential for reducing the prices of the medicines, the total exclusion becomes unnecessary, and therefore rare [25].

Conversely, in France, the most active country in adopting the complete disinvestment strategy, a comprehensive review of all 4,490 drugs listed between 2000 and 2004 was conducted. As a result, 835 drugs were excluded from the national list (drugs surpassed by new and more effective ones, unsafe medicines, and those no longer considered effective) and 617 drugs had a reduction of reimbursement rate from 65 to 35%. There were contestations by the industry and between 2003 and 2006, a reevaluation of 723 drugs that were previously withdrawn from the list confirmed that 525 of them were insufficient [25].

In Latin America, Agirrezabal et al. named some local disinvestment initiatives, although most of them did not present a comprehensive description of the process used in finding, prioritizing, evaluating, implementing, and the results of the disinvestment. Four studies referred to Brazilian cases, and only one was considered as a potential disinvestment approach [26]. In this case, Conitec assessed the intramuscular interferon beta-1a performance for multiple sclerosis. This analysis comprised two studies: a meta-analysis with mixed treatment comparison and an assessment of the efficacy of the medicine in Brazilian patients (12,154 patients) using the SUS database. The results demonstrated a statistically higher risk of treatment failure with intramuscular interferon beta - 1a (exchange of treatment or relapse or death), pointing to the inferiority of this medicine when compared to another interferon beta. Accordingly, there was the decision of restriction of use in the treatment of multiple sclerosis, with the possibility of maintenance of treatment for those who were already using it [27]. However, in October 2017, the use restriction was revoked without published justification, after an appeal by the company that holds the registration in Brazil [28].

Such results illustrate and emphasize the difficulty in removing technologies already available in the healthcare system, making it necessary to combine scientific data, in these cases, more detailed and extensive, with the stakeholders' interests. However, generating data that contradict some practices is not always enough, since many physicians continue to administer treatments proven ineffective and even harmful to patients [29].

In this context, policymakers may play a key role in regulating the use of such technologies, and therefore should consider the adoption of active forms of disinvestment, not only those aimed at total exclusion, but also the restriction treatment to subgroups, price or reimbursement rate reductions, encouragement of generic prescribing [25].

Regarding the indication of requests, the vast majority of the technologies reviewed were related to rheumatoid arthritis, HIV/AIDS, and hepatitis, which, along with oncology, represent the leading health topics for new adoption requests [30]. Considering the broad range of pathologies and conditions covered by SUS, there is a greater focus in a few diseases, very likely by the updates of the respective clinical protocols. However, many technologies are indicated to a non-covered condition by the clinical protocols and therapeutic guidelines, which may represent a gap in the selection process for reassessment. One example of this are the technologies for cancer treatment that are outside the scope of clinical protocols: 18 technologies were assessed during the period of this study, and none of them went through a review process. Two other technologies available in the health system, before the existence of the Committee, were reviewed–adjuvant chemotherapy, excluded for the treatment of squamous cell carcinoma and mammography, which received an unfavorable recommendation to the widening of the age range.

Thus, as highlighted above, reassessments were made based on demands, the vast majority presented by the Ministry of Health itself and departments or organizations linked to SUS. Demand-based assessment is limited by the fact that it does not systematize all technologies that need to be adjusted in the face of new evidence (scientific or contextual) and those that should be excluded because they are ineffective or unsafe.

Studies on disinvestment methodologies point out that the initial stages of selection and prioritization are fundamental to generate greater transparency and enable all technologies to be reviewed [12,31,32]. Some strategies can be applied in these stages, such as searching, monitoring and reviewing literature and public databases, consultation with clinical specialties groups, reports of HTA agencies, consulting the health service of the healthcare system, evaluation of variation in quantity (example 2, 3 or 5 years), amongst others [8,15,33].

However, setting the goal of the HTR program is required to decide on how to select and prioritize technologies, as Sustainability in Health care by Allocating Resources Effectively (SHARE) program in Australia advocates. In this case, "the aim of the SHARE Program was to establish organization-wide, systematic, integrated, transparent, evidence-based systems and processes for decision-making about disinvestment in the context of resource allocation at Monash Health" [34].

Other initiatives that have been implemented in Australia sought to refine the indications for resource allocation to ineffective or inappropriately applied health care practices, to subsidize decision-related to ineffective drugs and vaccines in the Pharmaceutical Benefits Scheme (PBS), also, to maximize the patients’ health and the supply sustainability. Some of these programs implemented in a local, regional and national level reported recommendations after RTS, such as: disinvestment of assisted reproduction technologies (in vitro fertilization and intracytoplasmic sperm injection), vitamin B12 and folate tests, discontinuation of public funding of kyphoplasty and percutaneous vertebroplasty for vertebral compression, revisions of colonoscopy items, surgical treatment for obesity, pulmonary artery catheterization, catheterization in ophthalmology, and restriction in the rules for screening for cervical cancer [35,36].

In Sweden, the nation-wide “Uncertainties and Disinvestment Project” was designed to identify scientific uncertainties, to inform and support decision makers in prioritizing and allocating resources within the health services. As a result, there was disinvestment of arthroscopy in arthritis, a corticosteroid to lateral epicondylitis and, clinical replacement versus routine replacement of peripheral venous catheters. In the UK, NICE recommendations based programmes aimed to inform about the financial aspect of resources utilization, safety, effectiveness and health care quality process. Another initiative in the UK intended to reconfigured services to provide more health gain at a reduced total cost [36].

Another point highlighted is the setting of deadlines for HTR, not observed in the Conitec process. Citing the example of other countries a recent systematic review of the sustainability programs and innovations have shown, in most of the studies, that the first evaluations were conducted after two years of the technology implementation [17,37]. The definition of fixed deadlines could eliminate the need for the selection of technologies for reassessment in the cases of Conitec appraisal for its adoption [33]. To those that entered the system without Conitec assessment, it would be necessary the adoption of standardized methodology with explicit criteria for screening the technologies to be reassessed to ensure periodic updates of all the list of healthcare technologies [31,32].

The dimensions of analysis found in the recommendation reports were scientific evidence on efficacy, effectiveness or safety, disease-related issues, issues related to the use of technology, costs, and social participation. However, these dimensions were not included in all analysis, and a standardized structure of the reports has not been observed.

To decision-making, the criteria of efficacy safety and use of the technology were the most relevant for the set of evaluations. These data are in conformance to the primary concern of the health systems from France, Australia, New Zealand, Canada and the UK, that is, to identify safety and efficacy problems that were not previously identified or underestimated in a randomized clinical trial [25]. Besides, the HTR has been used in others countries to assess the degree of adoption and usage of technology and, to provide the real costs and the use of health technologies resources in real settings [38].

Another critical point to be mentioned was the lack of social participation through public consultation in most of the decisions. Although the argument that some technologies can have a fast-track appraisal process, the rules for this type of evaluation are not well defined. Thus, such technologies were reassessed regardless of the stakeholders' value and preference, which may compromise the legitimacy and acceptability of the decision. In order to achieve greater acceptance of decisions and to identify the potential impacts in the society, HTR process could involve the politicians, clinicians, speciality societies, health system leaders, industry, and patients [33]. In the United Kingdom, for example, the Technology Appraisal Committee (TAC) which is responsible for decision making on disinvestment, has appointed members from the National Health Service (NHS), patient and care organizations, academia, pharmaceutical and medical device industries. In Australia, deliberative methods were adopted to develop stakeholder evidence-informed engagements. To achieve this, clinicians, consumers and representative members of the community participate in the assessment [39].

The decision-making oriented by the results of the technology performance, although not being observed as a regular practice by CONITEC, in other countries, some strategies have been adopted in this sense. Negotiation for price reduction and reduction of reimbursement rates has usually been implemented in countries such as Canada, France, Australia and New Zealand after analyzing the results of technology performance [25,39]. In a study carried out in Italy, the implementation of a product-specific monitoring registry was the possible determinant associated with a higher price reduction of proposed price and negotiated price [40].

Managed entry agreements (MEAs) contractual agreements also have been used when uncertainty regarding the clinical effectiveness, safety or resource use associated with a technology is high. This kind of agreement distributes the risk of uncertainty between the payers and the manufacturer. In this case, additional further evidence generation is required [41]. Following this trend, the Brazilian Ministry of Health recently held the first international risk-sharing workshop, thus beckoning to use performance data to adjust the price of technology [42]. This new scenario reinforces the need to structure and strengthen the system to monitor and evaluate the technologies available in the Brazilian health system, in order to evaluate them in real conditions of the practical clinic and consequently, attribute the real value of such technologies.

As limitations to the present study, we point out the reclassification of the decision types by the authors as there is no definition of the term "reassessment" in SUS, and this may not reflect the perspective of the technicians and managers who participate in the Conitec deliberations. Moreover, the arguments described in the reports might not explicitly portray the justifications for the decision-making presented during the Committee meeting.

## Conclusions

The reassessment process has appeared among other strategies for reducing the use of ineffective, less effective or unsafe practices and, consequently, to avoid a resources waste. The results of Conitec six years of work presented initiatives with this goal. Although positive results were obtained, this activity is not yet structured, with gaps regarding the selection phase and reassessment of SUS list of technologies, since it has not been identified method and criteria for its conduction.

Therefore, this study reinforces the need to prioritize structuring actions in within the SUS that improve the management of technologies, both those recently incorporated and also these adopted before the establishment of the Committee. Thus, it is suggested the implantation of a monitoring program and HTR, with endorsed methodology and that it contemplates in deepened way implementation questions (barriers and facilitators), stakeholder perspectives, and scientific, social, ethical, and legal questions. Such actions need, inevitably, short-term time and resources, but might add long-term gains in efficiency [43,44].

Finally, we expect that this study can contribute to the formulation of a systematic, transparent, and objective process for a revision of the list of technologies offered by the Brazilian Public Health System–SUS. Such process can be helpful in defining the "true" value of a given technology in real conditions of use, contributing to better quality in the healthcare and sustainability of the healthcare system.

## Supporting information

S1 Table Technologies assessed by Conitec from January 2012 until November 2017. (XLSX)

## References

[1] Kaló Z, Landa K, Doležal T, Vokó Z. Transferability of National Institute for Health and Clinical Excellence recommendations for pharmaceutical therapies in oncology to Central-Eastern European countries. Eur J Cancer Care (Engl). 2012;21(4):442–9. 22510226 10.1111/j.1365-2354.2012.01351.x

[2] Maison P, Zanetti L, Solesse A, Bouvenot G, Massol J, ISPEP group of the French National Authority for Health. The public health benefit of medicines: how it has been assessed in France? The principles and results of five years’ experience. Health Policy. 2013;112(3):273–84. 10.1016/j.healthpol.2013.04.007 23664299

[3] Kelly RJ, Smith TJ. Delivering maximum clinical benefit at an affordable price: engaging stakeholders in cancer care. Lancet Oncol. 2014;15(3):e112–8. 10.1016/S1470-2045(13)70578-3 24534294

[4] IBGE—Instituto Brasileiro de Geografia e Estatística. Conta-satélite de saúde: Brasil: 2010-2015/IBGE [Internet]. Rio de Janeiro, 2017. https://biblioteca.ibge.gov.br/visualizacao/livros/liv101437.pdf. Accessed 12 Dec 2017.

[5] Guindo LA, Wagner M, Baltussen R, Rindress D, Van Til J, Kind P, et al From efficacy to equity: Literature review of decision criteria for resource allocation and healthcare decisionmaking. Cost Eff Resour Alloc. 2012;10(1):9 10.1186/1478-7547-10-9 22808944 PMC3495194

[6] Marsh K, Lanitis T, Neasham D, Orfanos P, Caro J. Assessing the value of healthcare interventions using multi-criteria decision analysis: a review of the literature. Pharmacoeconomics. 2014;32(4):345–65. 10.1007/s40273-014-0135-0 24504851

[7] Kolasa K, Zwolinski KM, Kalo Z, Hermanowski T. Potential impact of the implementation of multiple-criteria decision analysis (MCDA) on the Polish pricing and reimbursement process of orphan drugs. Orphanet J Rare Dis. 2016;11(1):23. 26965710 10.1186/s13023-016-0388-0 PMC4787054

[8] Gerdvilaite J. Nachtnebel A. Disinvestment. Overview of disinvestment experiences and challenges in selected countries. HTA-Projektbericht 57 [Internet]. 2011 http://eprints.hta.lbg.ac.at/926/ Accessed 24 May 2018.

[9] Noseworthy T, Clement F. Health technology reassessment: Scope, methodology, & language. International Journal of Technology Assessment in Health Care. 2012;28(3):201–2. 10.1017/S0266462312000359 22980695

[10] Colla CH, Mainor AJ, Hargreaves C, Sequist T, Morden N. Interventions Aimed at Reducing Use of Low-Value Health Services: A Systematic Review. Med Care Res Rev. 2017;74(5):507–50. 10.1177/1077558716656970 27402662

[11] Prasad V, Vandross A, Toomey C, Cheung M, Rho J, Quinn S, et al A decade of reversal: an analysis of 146 contradicted medical practices. Mayo Clin Proc. 2013;88(8):790–8. 10.1016/j.mayocp.2013.05.012 23871230

[12] Niven DJ, Mrklas KJ, Holodinsky JK, Straus SE, Hemmelgarn BR, Jeffs LP, et al Towards understanding the de-adoption of low-value clinical practices: a scoping review. BMC Med. 2015;13(1):255. 26444862 10.1186/s12916-015-0488-z PMC4596285

[13] Sermet C, Andrieu V, Godman B, Van Ganse E, Haycox A, Reynier J-P. Ongoing pharmaceutical reforms in France: implications for key stakeholder groups. Appl Health Econ Health Policy. 2010;8(1):7–24. 10.2165/11313900-000000000-00000 20038190

[14] Garner S, Littlejohns P. Disinvestment from low value clinical interventions: NICEly done? BMJ (Online). 2011; 343: d4519–d4519. 10.1136/bmj.d4519 21795239

[15] Ruano-Ravina A, Varela-Lema L, Cerda-Mota T, Ibargoyen-Roteta N, Gutiérrez-Ibarluzea I, Blasco JA, et al Identificación, priorización y evaluación de tecnologías obsoletas. Guía metodológica. Plan de Calidad para el Sistema Nacional de Salud del Ministerio de Sanidad y Política Social. Axencia de Avaliación de Tecnoloxías Sanitarias de Galicia; 2007 Informes de Evaluación de Tecnologías Sanitarias: avalia-t No. 2007/01. https://www.sergas.es/docs/Avalia-t/ObsoleteTechMemFinal.pdf. Accessed 24 november 2017.

[16] Harris C, Allen K, King R, Ramsey W, Kelly C, Thiagarajan M. Sustainability in Health care by Allocating Resources Effectively (SHARE) 2: identifying opportunities for disinvestment in a local healthcare setting. BMC Health Serv Res. 2017;17(1):328 10.1186/s12913-017-2211-6 28476159 PMC5420107

[17] Scheirer MA. Is Sustainability Possible? A Review and Commentary on Empirical Studies of Program Sustainability. Am J Eval. 2005;26(3):320–47.

[18] Brasil. Presidência da República, Subchefia para Assuntos Jurídicos. Lei no 12.401, de 28 de abril de 2011. Altera a Lei no 8.080, de 19 de setembro de 1990, para dispor sobre a assistência terapêutica e a incorporação de tecnologia em saúde no âmbito do Sistema Único de Saúde–SUS [Internet]. Brasília, DF; 2011. http://www.planalto.gov.br/ccivil_03/_ato2011-2014/2011/lei/l12401.htm. Accessed 15 Mar 2018.

[19] Brasil. Decreto no 7.646, de 21 de dezembro de 2011. Dispõe sobre a Comissão Nacional de Incorporação de Tecnologias no Sistema Único de Saúde e sobre o processo administrativo para incorporação, exclusão e alteração de tecnologias em saúde pelo Sistema Único de Saúde[Internet]. Diário Oficial [da] República Federativa do Brasil. 2011. http://www.planalto.gov.br/CCIVIL_03/_Ato2011-2014/2011/Decreto/D7646.htm. Accessed 27 Jun 2017.

[20] Brasil. Ministério da Saúde. Secretaria de Atenção à Saúde. Protocolos Clínicos e Diretrizes Terapêuticas em Oncologia. Carcinoma de Pulmão. [Internet]. Brasília: Ministério da Saúde. 2014; 171–82 p. http://bvsms.saude.gov.br/bvs/publicacoes/protocolos_clinicos_diretrizes_terapeuticas_v3.pdf. Accessed 23 Oct 2017.

[21] Brasil. Ministério da Saúde. Decisões sobre a incorporação de tecnologias no SUS [Internet]. Brasília: Ministério da Saúde 2014 http://conitec.gov.br/decisoes-sobre-incorporacao-ordem-alfabetica. Accessed 14 Nov 2017.

[22] Hézode C, de ledinghen V, Fontaine H, Zoulim F, Lebray P, Boyer N, et al. Daclatasvir plus sofosbuvir with or without ribavirin in genotype 3 patients from a large French multicenter compassionate use program [astract]. AASLD Liver Meeting 2015. San Francisco, November 13–17, 2015. Abstract n. 206.

[23] Welzel TM, Petersen J, Herzer K, Ferenci P, Gschwantler M, Wedemeyer H, et al Daclatasvir plus sofosbuvir, with or without ribavirin, achieved high sustained virological response rates in patients with HCV infection and advanced liver disease in a real-world cohort. Gut. 2016;65(11):1861–70. 10.1136/gutjnl-2016-312444 27605539 PMC5099229

[24] Yuba TY, Novaes HMD, de Soárez PC. Challenges to decision-making processes in the national HTA agency in Brazil: operational procedures, evidence use and recommendations. Heal Res Policy Syst. 2018;16(1):40. 10.1186/s12961-018-0319-8 PMC5948855 29751764

[25] Parkinson B, Sermet C, Clement F, et al Disinvestment and Value-Based Purchasing Strategies for Pharmaceuticals: An International Review. Pharmacoeconomics 2015; 33: 905–24. 10.1007/s40273-015-0293-8 26048353

[26] Agirrezabal I, Burgon J, Stewart G, Gutierrez-Ibarluzea I. Status of disinvestment initiatives in Latin America: Results from a systematic literature review and a questionnaire. Int J Technol Assess Health Care. 2017;33(6):674–80. 10.1017/S0266462317000812 28956521

[27] Lemos LLP de Guerra Júnior AA, Santos M, Magliano C, Diniz I, Souza K, et al The Assessment for Disinvestment of Intramuscular Interferon Beta for Relapsing-Remitting Multiple Sclerosis in Brazil. Pharmacoeconomics. 2018;36(2):161–73. 10.1007/s40273-017-0579-0 29139001 PMC5805817

[28] Brasil. Ministério da Saúde. Portaria no 44 de 19 de outubro de 2017. Revoga a Portaria no 27, de 6 de julho de 2016, que restringiu o uso da betainterferona intramuscular 1A 6.000.000 UI (30 mcg) no tratamento da esclerose múltipla do subtipo remitente recorrente no âmbito do Sistema Único de Saúde—SUS. Diário Oficial [da] República Federativa do Brasil. 20 Oct 2017; Seção 1.p 176.

[29] Epstein D. and Publica P. When evidence says no, but doctors say yes. Atlantic. February, 22. https://www.theatlantic.com/health/archive/2017/02/when-evidence-says-no-but-doctors-say-yes/517368/. Accessed 20 5 2019.

[30] Rabelo RB, Petramale CA, Silveira LC da, Santos VCC, Gonçalves HC. A comissão nacional de incorporação de tecnologias no SUS: um balanço dos primeiros anos de atuação. Rev Eletronica Gestão Saúde. 2015;6(4):3225.

[31] Ibargoyen-Roteta NG-II, Asua J. Report on the development of the GuNFT Guideline. Guideline for Not Funding existing health Technologies in health care systems. Quality Plan for the NHS of the MHSP. Basque Office for Health Technology Assessment (Osteba). 2009. Health Technology Assessment Reports: OSTEBA N° 2007/11.

[32] Mayer J, Nachtnebel A. Disinvesting from ineffective technologies: lessons learned from current programs. Int J Technol Assess Health Care. 2016;31(6):355–62. 10.1017/S0266462315000641 26694654

[33] Maloney MA, Schwartz L, O’Reilly D, Levine M. Drug disinvestment frameworks: Components, challenges, and solutions. Int J Technol Assess Health Care. 2017;33(2):261–9. 10.1017/S0266462317000277 28703087

[34] Harris C, Green S, Ramsey W, Allen K, King R. Sustainability in Health care by allocating resources effectively (SHARE) 1: introducing a series of papers reporting an investigation of disinvestment in a local healthcare setting. BMC Health Serv Res 2017; 17: 323 10.1186/s12913-017-2210-7 28472962 PMC5418706

[35] Polisena J, Clifford T, Elshaug AG, Mitton C, Russell E, Skidmore B. Case studies that illustrate disinvestment and resource allocation decision-making processes in health care: a systematic review. Int J Technol Assess Health Care 2013; 29: 174–84. 10.1017/S0266462313000068 23514665

[36] Orso M, de Waure C, Abraha I, et al Health technology disinvestment worldwide: overview of programs and possible determinants. Int J Technol Assess Health Care 2017; 33: 239–50. 10.1017/S0266462317000514 28669355

[37] Stirman SW, Kimberly J, Cook N, Calloway A, Castro F, Charns M. The sustainability of new programs and innovations: a review of the empirical literature and recommendations for future research. Implement Sci. 2012;7(1):17. 22417162 10.1186/1748-5908-7-17 PMC3317864

[38] Varela-Lema L, Ruano-Ravina A, Mota TC, et al Post-introduction observation of healthcare technologies after coverage: the Spanish proposal. Int J Technol Assess Health Care. 2012; 28:285–93. 10.1017/S0266462312000232 22980706

[39] Seo H-J, Park JJ, Lee SH. A systematic review on current status of health technology reassessment: insights for South Korea. Heal Res policy Syst 2016; 14: 82. 10.1186/s12961-016-0152-x PMC5106773 27835964

[40] Villa F, Tutone M, Altamura G, Antignani S, Cangini A, Fortino I, Melazzini M, Trotta F, Tafuri G, Jommi C, Determinants of price negotiations for new drugs. The experience of the Italian Medicine Agency. Health policy. 2019:123(6):595–600. 10.1016/j.healthpol.2019.03.009 31097207

[41] Brugger U. A review of Coverage with Evidence Development (CED) in different countries: what works and what doesn't [Internet]. Quebec: Health Technology Assessment International. 2014. https://htai.org/wp-content/uploads/2018/02/CED_Report_Bruegger_Final_Version.pdf. Accessed 19 May 2019.

[42] Brasil, Ministério da Saúde. Workshop de compartilhamento de risco. http://conitec.gov.br/workshop-de-compartilhamento-de-risco Accessed 15 May 2019.

[43] Leggett L, Noseworthy TW, Zarrabi M, Lorenzetti D, Sutherland LR, Clement FM. Health technology reassessment of non-drug technologies: Current practices. Int J Technol Assess Health Care. 2012;28(3):220–7. 10.1017/S0266462312000438 22980697

[44] Guerra-Júnior AA, Pires De Lemos LL, Godman B, Bennie M, Osorio-De-Castro CGS, Alvares J, et al Health technology performance assessment: Real-world evidence for public healthcare sustainability. Int J Technol Assess Health Care. 2017;33(2):279––87. 10.1017/S0266462317000423 28641588

